# Association of coffee and tea consumption with the risk of atrial fibrillation: a prospective cohort study from the UK Biobank

**DOI:** 10.29219/fnr.v70.14612

**Published:** 2026-10-31

**Authors:** Rui Liang, Zhaofeng Zhang, Yacong Bo, Jing Guo

**Affiliations:** 1 Department of Nutrition, The First Affiliated Hospital of Zhengzhou University, Zhengzhou, China; 2 Department of Nutrition and Food Hygiene, School of Public Health, Peking University, Beijing, People’s Republic of China; 3 Beijing’s Key Laboratory of Food Safety Toxicology Research and Evaluation, Beijing, People’s Republic of China; 4 Department of Nutrition and Food Hygiene, College of Public Health, Zhengzhou University, Zhengzhou, China; 5 Department of Kinesiology, School of Physical Education (Main Campus), Zhengzhou University, Zhengzhou, China

**Keywords:** coffee intake, tea intake, atrial fibrillation, cohort study, UK Biobank

## Abstract

**Objectives:**

Atrial fibrillation (AF) has a high prevalence and a heavy healthcare burden, making prevention of AF essential. Coffee and tea are popular beverages around the world, and they may have a relationship with AF, but the results are controversial. This study aimed to investigate the relationship of coffee and tea intake with the risk of AF.

**Methods:**

This study included 495,269 participants without AF at baseline from the UK Biobank. Coffee and tea intake was self-reported (0, 0.5–1, 2–3, ≥ 4 cups/day). Cox regression was used to estimate hazard ratios and 95% confidence intervals.

**Results:**

During a median follow-up of 13.04 ± 2.26 years, 37,261 participants developed AF. The associations of coffee and tea with AF were non-linear (P for non-linear < 0.001). Compared with non-drinkers, drinking 0.5–1, 2–3, and ≥ 4 cups of coffee per day was associated with a protective effect against AF, corresponding to a 7, 11, and 9% lower risk, respectively. For tea, only consumption of 2–3 cups per day was associated with a modestly lower risk of AF, whereas no significant associations were observed for other intake categories.

**Conclusions:**

We found that coffee intake was inversely associated with incident AF across multiple intake categories, whereas for tea, a modest inverse association was observed only at 2–3 cups per day, with no statistically significant associations for other intake categories. Further studies are warranted to confirm our findings and clarify the potential mechanism.

## Popular scientific summary

Coffee intake (0.5–1, 2–3, ≥ 4 cups/day) was associated with 7, 11, and 9% lower AF risk; tea only showed a 5% reduction at 2–3 cups/day. Moderate coffee or tea intake may be associated with a lower risk of AF. Causal confirmation and mechanistic studies are needed.

Atrial fibrillation (AF) has long been regarded as the most common arrhythmia. Approximately 33 million people worldwide are affected by AF, and morbidity and mortality due to AF are increasing annually (1). AF is a condition in which the heart is subjected to abnormal electrical impulses that prevent it from beating at its normal rhythm, resulting in a variety of symptoms including irregular heart rate, palpitations and shortness of breath (2). Costs directly related to AF treatment account for 1% of total healthcare support in the UK, while the cost of AF-related complications and treatments reaches $26 billion in the U.S. Without timely and effective prevention and treatment of AF, these costs will increase dramatically (3, 4). Given the substantial healthcare expenses associated with AF treatment techniques, it is important to pinpoint modifiable risk factors for AF and implement effective preventive strategies. A study has suggested that multiple dietary components may reduce the risk of AF, such as caffeine (5).

Coffee and tea, the most widely consumed beverages in the world besides water, are the main sources of caffeine intake. Caffeine is a naturally occurring central nervous system stimulant and has long been suspected to exert adverse cardiovascular effects because of its stimulatory properties (6). However, accumulating evidence indicates that moderate consumption of caffeinated beverages, particularly coffee and tea, is generally safe and may even confer cardiovascular benefits (7, 8). In contrast, the relationship between coffee and tea intake and AF remains uncertain. Previous meta-analyses have reported inconsistent findings, with some suggesting a higher AF risk, whereas others observed no significant association or even a lower AF risk with moderate coffee consumption (9, 10). However, many previous studies were limited by relatively small sample sizes, insufficient control for potential confounding, restricted population representativeness, and limited evaluation of dose–response associations. To address these research gaps, we conducted a large prospective cohort study using UK Biobank data, which includes both men and women, detailed assessments of coffee and tea intake, and comprehensive covariate information, allowing a more robust evaluation of the associations between beverage intake and incident AF.

## Materials and methods

### Study population

The data analyzed in this study were based on the UK Biobank, which is a large prospective cohort of approximately 502,000 adults aged 40–69 years recruited from the general United Kingdom (UK) population between 2006 and 2010 (11). The study design of the UK Biobank has been previously described in detail (12). The UK Biobank received ethical approval from the North West Multi-centre Research Ethics Committee (REC reference: 21/NW/0157), and all participants provided written informed consent. The present analyses were conducted under UK Biobank application number 93398.

In this study, a total of 502,371 participants attended the UK Biobank medical examination between 2006 and 2010. We initially excluded 2,200 participants without data on coffee and tea intake, leaving 500,171 participants with complete beverage information. We further excluded 4,902 participants who had AF at baseline, resulting in 495,269 participants included in the final analysis. Figure 1 presents a flowchart of the participant selection process.

**Fig. 1:**
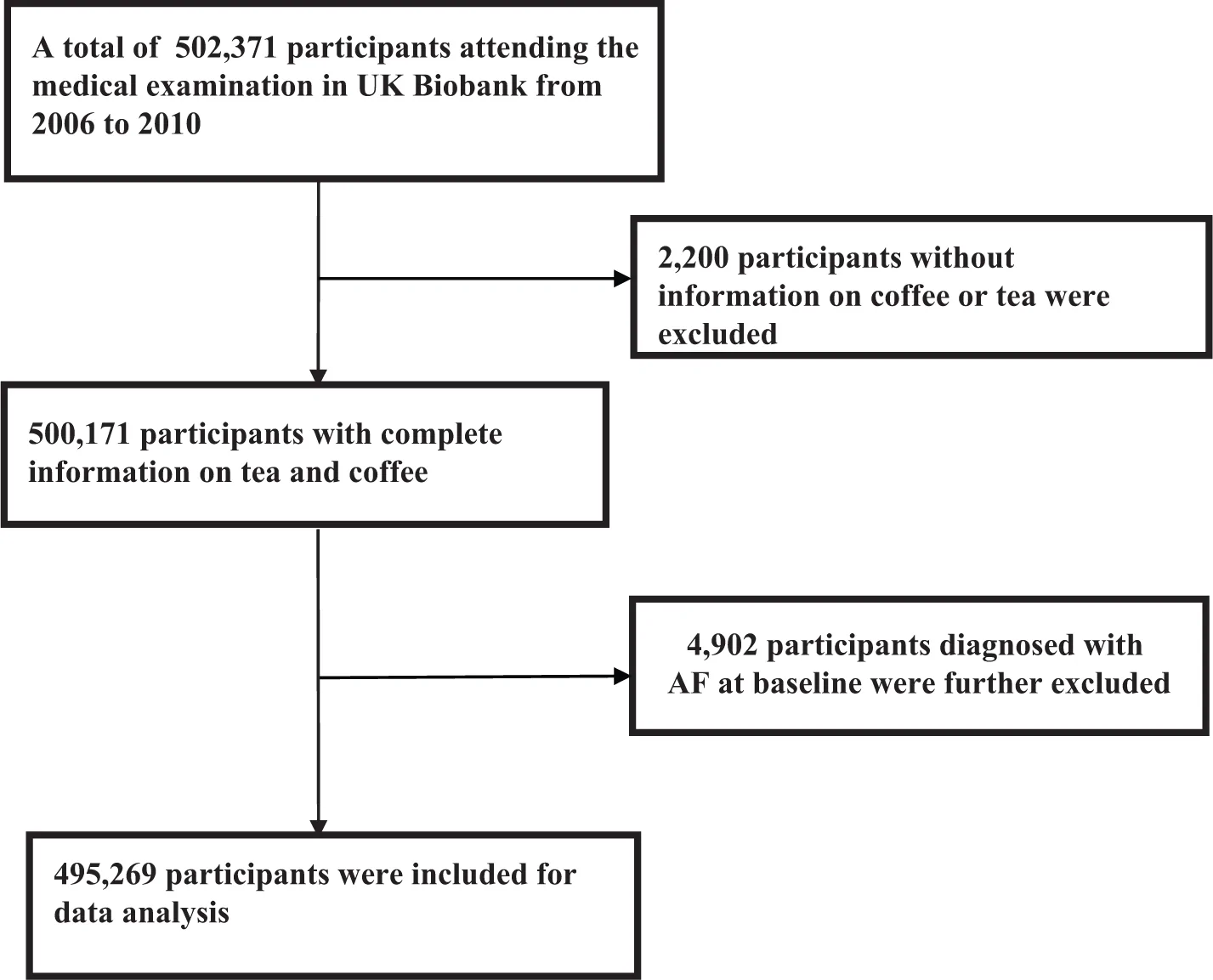
Flowchart of participant selection.

### Exposure assessment

The intake of coffee and tea was assessed by using a touchscreen questionnaire at baseline. During the process, participants were asked to make choices on the following questions: ‘How many cups of coffee do you drink each day? (including decaffeinated coffee)’; ‘How many cups of tea do you drink each day (including black and green tea)?’. Specific number of cups (0–9), ‘less than one’, ‘do not know’, or ‘prefer not to answer’ will be selected by participants. If the participants choose more than 10 cups, then the participants will be asked to identify their answer.

In this study, to explore the association between coffee and tea intake with AF, the coffee and tea intake was categorized into four groups: 0, 0.5–1, 2–3, and ≥ 4 cups per day. Participants with no coffee and tea consumed were included as respective control groups. Because the coffee intake question included decaffeinated coffee, the present analyses reflect overall coffee beverage consumption rather than caffeine exposure per se. Caffeine intake was not directly quantified in this study.

### Outcome ascertainment

The incident AF (ICD codes: I48) was the outcome of this study, defined by the three-character codes with the International Statistical Classification of Diseases and Related Health Problems 10th Revision (ICD-10). AF was diagnosed by either self-report at any assessment center, inpatient hospital data, primary care or death record data.

### Covariates

In order to ensure the robustness of the results, it is important to consider covariates related to demographic variables (sex, age, race, education level, income) as well as potential confounding factors that may be associated with AF, including townsend deprivation index, body mass index (BMI), physical activity (PA), alcohol drinking, and smoking status. Race was categorized as white and other; Education level included college or university degree, advanced (A) levels/Advanced Subsidiary (AS) levels or equivalent, Ordinary (O) levels/General Certificate of Secondary Education (GCSE) or equivalent, Certificate of Secondary Education (CSE) or equivalent, National Vocational Qualification (NVQ) or Higher National Diploma (HND) or Higher National Certificate (HNC) or equivalent, other professional qualifications, or none of the above; The income was divided into five categories: less than £18,000, £18,000 to £30,999, £31,000 to £51,999, £52,000 to £100,000, and greater than 100,000. PA was classified into three categories: low-level PA, moderate-level PA and high-level PA. Alcohol drinking and smoking status were divided into three categories, respectively, current, ever and never.

### Statistical analysis

The baseline characteristics were summarized into normal continuous variables expressed as means and standard deviation (SDs), non-normal continuous variables expressed as median and interquartile ranges (IQRs), and categorical variables expressed as percentages based on the categories of coffee and tea consumption. Missing values in covariates were imputed using multiple imputation by chained equations (MICE). Among 495,269 participants, the proportions of missing data were 19.6% for PA, 15.0% for income, 1.8% for education, 0.5% for BMI, 0.4% for smoking, 0.1% for alcohol consumption, 0.1% for Townsend deprivation index, and <0.01% for age; sex and ethnicity had no missing values. We generated five imputed datasets with five iterations. Variables imputed were age, PA, education, income, smoking, alcohol consumption, BMI, and Townsend deprivation index; sex and ethnicity were used as auxiliary variables but were not imputed. Continuous variables were imputed using predictive mean matching, and categorical variables using appropriate regression models. Cox models were fitted in each imputed dataset, and hazard ratios (HRs) were pooled using Rubin’s rules. To estimate the HRs and 95% confidence intervals (CIs) of separate coffee and tea consumption with the risk of AF, we used cox proportional hazards models. The proportional hazards assumption was assessed using Schoenfeld residuals, and no violations were found. In order to exclude the effect of covariates, we also adjusted for potential confounders including (age, sex, ethnic, PA, education level, income, smoking status, drinking, BMI, Townsend deprivation index). Restricted cubic splines were used to evaluate the dose–response relationships. In addition, subgroup analyses were used to assess potential effect modification and to determine whether population heterogeneity existed based on sex (male, female), age (< 65, ≥ 65), BMI (< 25, ≥ 25), and smoking status (never, ever). The robustness of the association of coffee and tea intake with incident AF was evaluated through sensitivity analyses that (1) excluded participants who developed AF within the first 2 years of follow-up, (2) excluded individuals with cardiovascular disease (CVD) at baseline, (3) excluded individuals with missing covariates, and (4) further adjusted for major AF-related clinical confounders, including hypertension, diabetes mellitus, sleep apnea, coronary artery disease, and the use of beta blockers and antiarrhythmic drugs. All *P*-values were two-sided, and statistical significance was set to less than 0.05. All analyses were performed using R (version 4.0.0, R Foundation for Statistical Computing).

## Results

### Demographic characteristics

A total of 495,269 participants (mean age: 57.01 ± 8.09, 54.6% female) were included in this study. Overall, 94.4% of participants were White. No-coffee drinkers were 109,936 (22.2%), and the number of participants who drank 0.5–1, 2–3, ≥ 4 cups of coffee per day was 135,171 (27.3%), 153,499 (31.0%), and 96,663 (19.5%), respectively. Compared to non-coffee drinkers, coffee drinkers are more likely to have the following characteristics: male, white, higher level of education, never drinking, ever smoking.

In terms of tea intake, 72,809 (14.7%) participants did not drink tea, while 57,098 (11.5%), 145,385 (29.4%), and 219,977 (44.4%) consumed 0.5–1, 2–3, and ≥ 4 cups of tea per day. The characteristics of participants who drink tea were more likely to be male, white, higher level of PA, never drinking, current smoking (Table 1).

**Table 1:** Demographic and lifestyle characteristics of 495,269 participants by coffee and tea intake category in the UK Biobank Study

Characteristics	Overall	Coffee				Tea			
		0	0.5–1	2–3	≥ 4	0	0.5–1	2–3	≥ 4
Number, *n* (%)	495,269	109,936 (22.2)	135,171 (27.3)	153,499 (31.0)	96,663 (19.5)	72,809 (14.7)	57,098 (11.5)	145,385 (29.4)	219,977 (44.4)
Mean age (SD), y	57.01 (8.09)	55.94 (8.22)	57.48 (8.05)	57.56 (8.00)	56.71 (7.98)	55.93 (8.23)	55.99 (8.34)	57.16 (8.13)	57.55 (7.88)
Sex, *n* (%)									
Female	270,309 (54.6)	64,210 (58.4)	76,781 (56.8)	82,299 (53.6)	47,019 (48.6)	41,221 (56.6)	29,873 (52.3)	79,120 (54.4)	120,095 (54.6)
Male	224,960 (45.4)	45,726 (41.6)	58,390 (43.2)	71,200 (46.4)	49,644 (51.4)	31,588 (43.4)	27,225 (47.7)	66,265 (45.6)	99,882 (45.4)
Race and ethnicity, *n* (%)									
White	467,290 (94.4)	98,760 (89.8)	126,451 (93.5)	147,707 (96.2)	94,372 (97.6)	69,385 (95.3)	52,260 (91.5)	133,848 (92.1)	211,797 (96.3)
Other	27,979 (5.6)	11,176 (10.2)	8,720 (6.5)	5,792 (3.8)	2,291 (2.4)	3,424 (4.7)	4,838 (8.5)	11,537 (7.9)	8,180 (3.7)
Education, *n* (%)									
College or University	162,516 (32.8)	30,005 (27.3)	46,127 (34.1)	55,796 (36.3)	30,588 (31.6)	22,323 (30.7)	23,086 (40.4)	51,188 (35.2)	65,919 (30.0)
A/AS levels	55,792 (11.3)	11,701 (10.6)	15,752 (11.7)	17,745 (11.6)	10,594 (11.0)	8,479 (11.6)	7,173 (12.6)	16,715 (11.5)	23,425 (10.6)
O levels/GCSEs	106,081 (21.4)	23,820 (21.7)	28,931 (21.4)	32,330 (21.1)	21,000 (21.7)	16,529 (22.7)	11,685 (20.5)	31,035 (21.3)	46,832 (21.3)
CSEs or equivalent	27,071 (5.5)	7,361 (6.7)	6,403 (4.7)	7,445 (4.9)	5,862 (6.1)	4,677 (6.4)	2,609 (4.6)	7,361 (5.1)	12,424 (5.6)
NVQ or HND or HNC	32,816 (6.6)	7,832 (7.1)	8,609 (6.4)	9,337 (6.1)	7,038 (7.3)	4,881 (6.7)	3,154 (5.5)	8,927 (6.1)	15,854 (7.2)
Other professional qualifications	25,925 (5.2)	5,578 (5.1)	7,124 (5.3)	8,171 (5.3)	5,052 (5.2)	3,724 (5.1)	2,687 (4.7)	7,461 (5.1)	12,053 (5.5)
None of the above	85,068 (17.2)	23,639 (21.5)	22,225 (16.4)	22,675 (14.8)	16,529 (17.1)	12,196 (16.8)	6,704 (11.7)	22,698 (15.6)	43,470 (19.8)
Median Townsend deprivation index (IQR)	-1.31 (3.08)	-0.86 (3.27)	-1.40 (3.03)	-1.54 (2.97)	-1.34 (3.06)	-1.31 (3.08)	-1.09 (3.17)	-1.38 (3.06)	-1.38 (3.04)
Income, *n* (%)									
< 18,000	121,781 (24.6)	31,918 (29.0)	33,604 (24.9)	33,711 (22.0)	22,548 (23.3)	18,135 (24.9)	11,697 (20.5)	33,193 (22.8)	58,756 (26.7)
18,000–30,999	128,026 (25.8)	28,316 (25.8)	35,441 (26.2)	39,764 (25.9)	24,505 (25.4)	18,668 (25.6)	13,712 (24.0)	37,428 (25.7)	58,218 (26.5)
31,000–51,999	125,596 (25.4)	26,648 (24.2)	33,754 (25.0)	40,098 (26.1)	25,096 (26.0)	18,717 (25.7)	15,120 (26.5)	37,302 (25.7)	54,457 (24.8)
52,000–100,000	95,027 (19.2)	18,877 (17.2)	25,522 (18.9)	31,067 (20.2)	19,561 (20.2)	13,842 (19.0)	12,609 (22.1)	29,324 (20.2)	39,252 (17.8)
> 100,000	24,839 (5.0)	4,177 (3.8)	6,850 (5.1)	8,859 (5.8)	4,953 (5.1)	3,447 (4.7)	3,960 (6.9)	8,138 (5.6)	9,294 (4.2)
Mean body mass index (SD), kg/m^2^	27.06 (4.58)	27.07 (4.82)	26.69 (4.49)	26.95 (4.41)	27.77 (4.65)	27.77 (5.05)	27.11 (4.67)	26.83 (4.45)	26.97 (4.45)
Physical activity, *n* (%)									
Low	93,789 (18.9)	22,159 (20.2)	24,335 (18.0)	27,416 (17.9)	19,879 (20.6)	15,069 (20.7)	11,292 (19.8)	26,850 (18.5)	40,578 (18.4)
Moderate	201,732 (40.7)	43,218 (39.3)	55,981 (41.4)	63,978 (41.7)	38,555 (39.9)	28,781 (39.5)	23,676 (41.5)	60,622 (41.7)	88,653 (40.3)
High	199,748 (40.3)	44,559 (40.5)	54,855 (40.6)	62,105 (40.5)	38,229 (39.5)	28,959 (39.8)	22,130 (38.8)	57,913 (39.8)	90,746 (41.3)
Drinking, *n* (%)									
Current	21,867 (4.4)	9,598 (8.7)	5,231 (3.9)	4,282 (2.8)	2,756 (2.9)	3,882 (5.3)	2,361 (4.1)	6,601 (4.5)	9,023 (4.1)
Ever	17,730 (3.6)	6,340 (5.8)	3,692 (2.7)	3,861 (2.5)	3,837 (4.0)	3,712 (5.1)	1,685 (3.0)	3,905 (2.7)	8,428 (3.8)
Never	455,672 (92.0)	93,998 (85.5)	126,248 (93.4)	145,356 (94.7)	90,070 (93.2)	65,215 (89.6)	53,052 (92.9)	134,879 (92.8)	202,526 (92.1)
Smoke, *n* (%)									
Current	271,568 (54.8)	64,738 (58.9)	78,385 (58.0)	84,084 (54.8)	44,361 (45.9)	38,019 (52.2)	31,200 (54.6)	82,469 (56.7)	119,880 (54.5)
Ever	171,386 (34.6)	34,462 (31.3)	46,545 (34.4)	55,159 (35.9)	35,220 (36.4)	24,803 (34.1)	19,665 (34.4)	50,744 (34.9)	76,174 (34.6)
Never	52,315 (10.6)	10,736 (9.8)	10,241 (7.6)	14,256 (9.3)	17,082 (17.7)	9,987 (13.7)	6,233 (10.9)	12,172 (8.4)	23,923 (10.9)

Note: Data are presented for descriptive purposes only; no statistical comparisons were performed between intake categories. For categorical variables, data are shown as *n* (%), with percentages calculated using the number of participants in the corresponding column as the denominator. For continuous variables, data are shown as mean (SD). Coffee and tea intake categories were analyzed separately; participants could belong to different intake categories for coffee and tea. A/AS: advanced levels/Advanced Subsidiary; GCSC: General Certificate of Secondary Education; CSE: Certificate of Secondary Education; NVQ: National Vocational Qualification; HND: Higher National Diploma; HNC: Higher National Certificate; IQR: interquartile ranges; SD: standard deviation.

### Association of coffee and tea intake with incident AF

The coffee and tea consumption were classified into the four categories: 0, 0.5–1, 2–3, and ≥4 cups per day, and the association of these categories with the incident AF was examined (Table 2). After multivariate adjustment, coffee intake across 0.5–1, 2–3, and ≥4 cups per day was associated with a lower risk of AF. For tea, a statistically significant association was observed only among participants who consumed 2–3 cups per day. Compared with non-coffee drinkers, the risk of AF was reduced by 7% (HR 0.93, 95% CI, 0.9–0.96), 11% (HR 0.89, 95% CI, 0.86–0.92), and 9% (HR 0.91, 95% CI, 0.89–0.94) among those who drank 0.5–1, 2–3, and ≥4 cups per day, respectively. Compared with non-tea drinkers, those who drank 2–3 cups of tea per day had a 5% reduced risk of AF (HR 0.95, 95% CI 0.92–0.98). However, participants who drank 0.5–1 and ≥4 cups of tea per day had no association with the risk of AF (HR 0.96, 95% CI, 0.92–1.00), (HR 0.97, 95% CI, 0.94–1.00).

**Table 2:** HRs and 95% CIs for atrial fibrillation by coffee and tea intake among the 495,269 persons in the UK Biobank Study

	Coffee							Tea						
	0	0.5–1	*P*	2–3	*P*	≥ 4	*P*	0	0.5–1	*P*	2–3	*P*	≥ 4	*P*
Number	109,936	135,171		153,499		96,663		72,809	57,098		145,385		219,977	
Case, *N*	8,135	10,306		11,526		7,294		5,192	3,909		10,770		17,390	
Model 1^a^	Ref	1.03 (1.00–1.06)	0.042	1.01 (0.98–1.04)	0.397	1.02 (0.99–1.06)	0.151	Ref	0.95 (0.91–0.99)	0.021	1.03 (1.00–1.07)	0.057	1.11 (1.08–1.15)	< 0.001
Model 2^b^	Ref	0.88 (0.86–0.91)	< 0.001	0.85 (0.82–0.87)	< 0.001	0.92 (0.89–0.95)	< 0.001	Ref	0.90 (0.86–0.94)	< 0.001	0.89 (0.86–0.92)	< 0.001	0.94 (0.91–0.97)	< 0.001
Model 3^c^	Ref	0.93 (0.90–0.96)	< 0.001	0.89 (0.86–0.92)	< 0.001	0.91 (0.89–0.94)	< 0.001	Ref	0.96 (0.92–1.00)	0.032	0.95 (0.92–0.98)	0.002	0.97 (0.94–1.00)	0.025

a Original model without adjusting for any variables.

b Adjusted for age and sex.

c Adjusted for age, sex, ethnic, PA, education, income, smoking, drinking, BMI, and Townsend deprivation index. HR: hazard ratio; CI: confidence interval; PA: physical activity; BMI: body mass index.

### Nonlinear association

The restricted cubic splines were used to assess the relationship between coffee and tea intake with AF. After multiple adjustment modeling, non-linear associations were observed for both coffee and tea (*P* for non-linearity < 0.001 for both; Fig. 2). Specifically, intake of 2–3 cups coffee or tea per day was associated with the lowest HR for incident AF.

**Fig. 2:**
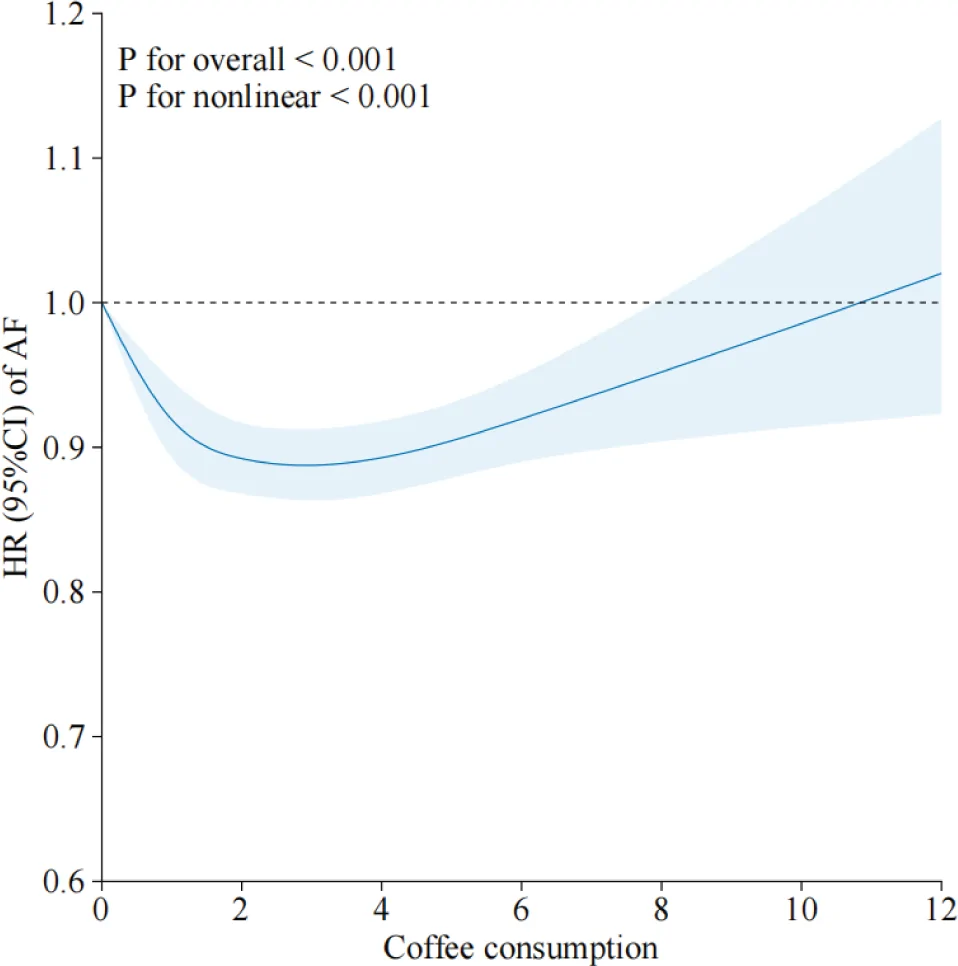
Does-response between coffee consumption and risk of AF. HR: hazard ratio; CI: confidence interval; AF: atrial fibrillation.

**Fig. 3:**
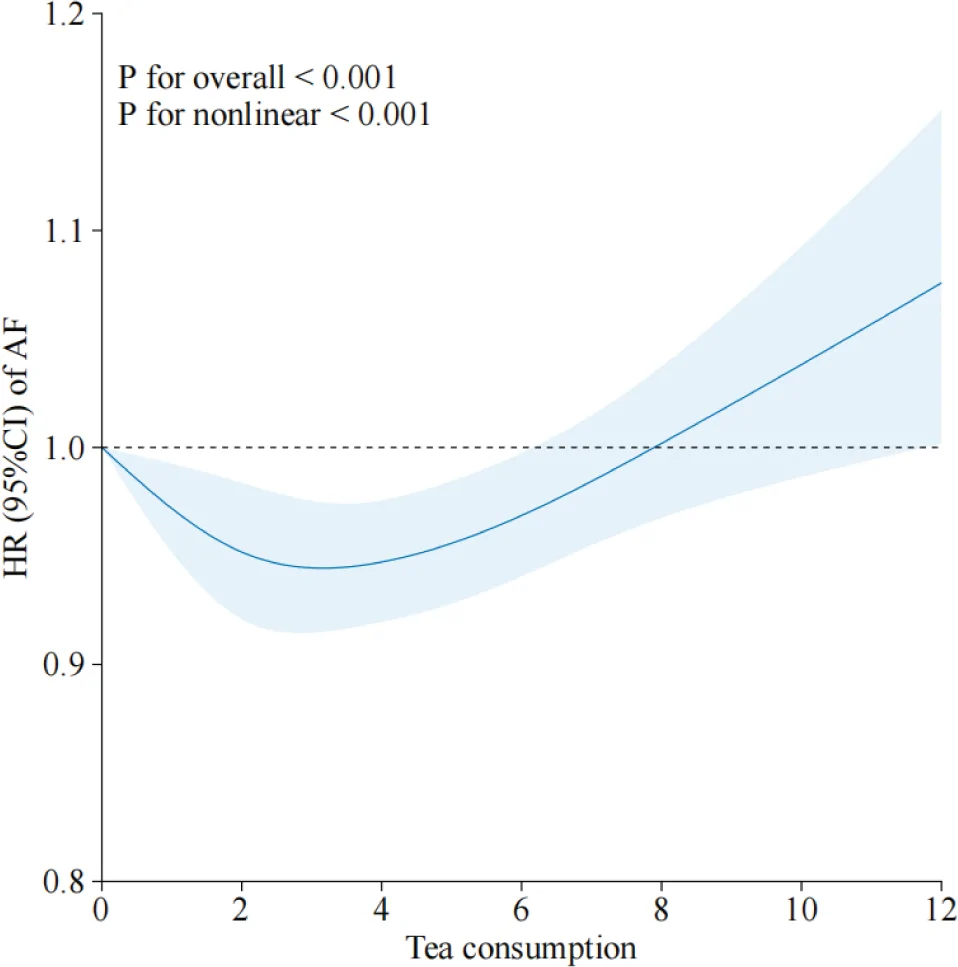
Does-response between tea consumption and risk of AF. HR: hazard ratio; CI: confidence interval; AF: atrial fibrillation.

### Subgroup analysis and sensitivity analysis

Subgroup analyses were conducted to examine whether the associations of coffee and tea intake with incident AF differed across sex, age, smoking status, and BMI. Multiplicative interaction tests were performed to assess whether effect estimates differed significantly between subgroups. No significant effect modification was observed (all *P* for interaction > 0.05; Table 3). To evaluate the robustness of the primary findings, we performed several sensitivity analyses. The associations of coffee and tea intake with incident AF remained consistent after excluding participants who developed AF within the first 2 years of follow-up, those with baseline CVD, and those with missing covariates. Similar findings were observed when the models were further adjusted for major AF-related clinical confounders, including hypertension, diabetes mellitus, sleep apnea, coronary artery disease, and use of beta blockers and antiarrhythmic drugs (Table 4).

**Table 3:** Stratified analysis HRs and 95% CIs for atrial fibrillation by coffee and tea intake among 495,269 persons in the UK Biobank Study

Characteristics	Coffee					Tea				
	0	0.5–1	2–3	≥ 4	Pinteraction	0	0.5–1	2–3	≥ 4	Pinteraction
Sex					0.982					0.950
Male	Ref	0.91 (0.87–0.94)	0.89 (0.85–0.92)	0.89 (0.85–0.93)		Ref	0.96 (0.91–1.01)	0.96 (0.92–1.00)	0.97 (0.93–1.01)	
Female	Ref	0.96 (0.92–1.01)	0.89 (0.85–0.93)	0.94 (0.89–0.99)		Ref	0.95 (0.89–1.02)	0.94 (0.89–0.99)	0.97 (0.92–1.01)	
Age					0.301					0.215
< 65	Ref	0.93 (0.90–0.97)	0.9 (0.87–0.93)	0.92 (0.88–0.96)		Ref	0.98 (0.93–1.03)	0.95 (0.91–0.99)	0.96 (0.92–1.00)	
≥ 65	Ref	0.92 (0.88–0.96)	0.87 (0.83–0.91)	0.89 (0.85–0.94)		Ref	0.92 (0.86–0.99)	0.95 (0.9–1.00)	0.98 (0.93–1.03)	
Smoker					0.547					0.194
Never	Ref	0.95 (0.91–0.99)	0.91 (0.87–0.95)	0.91 (0.87–0.96)		Ref	0.99 (0.93–1.06)	0.97 (0.92–1.02)	0.99 (0.94–1.04)	
Ever	Ref	0.92 (0.88–0.96)	0.88 (0.84–0.91)	0.92 (0.88–0.96)		Ref	0.93 (0.88–0.98)	0.93 (0.89–0.97)	0.95 (0.91–0.99)	
BMI					0.511					0.116
< 25	Ref	0.93 (0.88–0.99)	0.88 (0.83–0.94)	0.91 (0.85–0.98)		Ref	0.97 (0.89–1.07)	0.96 (0.89–1.03)	0.98 (0.92–1.05)	
≥ 25	Ref	0.93 (0.90–0.96)	0.89 (0.86–0.92)	0.91 (0.88–0.95)		Ref	0.95 (0.91–1.00)	0.95 (0.91–0.98)	0.96 (0.93–1.00)	

HR: hazard ratio; CI: confidence interval; BMI: body mass index.

**Table 4:** Sensitivity analysis HRs and 95% CIs for atrial fibrillation by coffee and tea intake among the 495,269 persons in the UK Biobank Study

Characteristics	Coffee				Tea			
	0	0.5–1	2–3	≥ 4	0	0.5–1	2–3	≥ 4
Excluding atrial fibrillation participants diagnosed within 2 years after baseline								
Number of participants	109,666	134,823	153,124	96,434	72,637	56,971	145,026	219,413
Number of case	7,865	9,958	11,151	7,065	5,020	3,782	10,411	16,826
Adjusted HR (95% CI)	Ref	0.93 (0.90–0.96)	0.89 (0.86–0.92)	0.92 (0.89–0.95)	Ref	0.96 (0.92–1.00)	0.95 (0.92–0.98)	0.97 (0.94–1.00)
Excluding individuals with cardiovascular disease at baseline								
Number of participants	76,093	94,693	109,267	68,810	51,413	41,073	102,707	153,670
Number of case	3,525	4,877	5,715	3,575	2,427	1,895	5,097	8,273
Adjusted HR (95% CI)	Ref	0.96 (0.92–1.01)	0.93 (0.89–0.97)	0.95 (0.91–1.00)	Ref	0.95 (0.89–1.00)	0.94 (0.89–0.98)	0.98 (0.93–1.02)
Excluding individuals with missing covariates								
Number of participants	74,410	95,154	110,167	68,827	51,204	41,424	103,149	152,781
Number of case	5,191	6,967	8,009	4,995	3,518	2,725	7,370	11,549
Adjusted HR (95% CI)	Ref	0.94 (0.90–0.97)	0.90 (0.87–0.94)	0.92 (0.89–0.96)	Ref	0.94 (0.90–0.99)	0.94 (0.90–0.98)	0.96 (0.92–0.99)
Adjusted for major AF-related clinical confounders								
Number of participants	109,936	135,171	153,499	96,663	72,809	57,098	145,385	219,977
Number of case	8,135	10,306	11,526	7,294	5,192	3,909	10,770	17,390
Adjusted HR (95% CI)	Ref	0.94 (0.91–0.97)	0.90 (0.88–0.93)	0.93 (0.90–0.96)	Ref	0.96 (0.93–1.01)	0.96 (0.93–0.99)	0.97 (0.94–1.01)

HR: hazard ratio; CI: confidence interval; AF: atrial fibrillation.

## Discussion

In this prospective analysis of 495,269 UK Biobank participants followed for 13.04 ± 2.26 years, we found that coffee intake was associated with a lower risk of AF across multiple intake categories, with the strongest association observed at 2–3 cups per day (11% lower risk). For tea, only consumption of 2–3 cups per day was associated with a modestly lower risk of AF (5% lower risk), whereas no statistically significant associations were observed for 0.5–1 or ≥4 cups per day.

It has long been suggested that coffee consumption may increase the risk of CVD (13). There are studies that suggest the above point, and an increase in coffee consumption is associated with an increased risk of AF (14). However, other research has revealed a totally different view. A prospective cohort study from a Danish study showed that coffee consumption was not associated with AF (15). There has been evidence about the health benefits of drinking coffee, a review of coffee and CVD suggested that coffee and tea, when consumed in moderation, do not appear to be harmful and may even be beneficial for a range of cardiovascular conditions, such as AF (16). Our findings are consistent with this review, supporting that coffee intake is associated with a lower risk of AF. Similarly, the relationship between tea intake and AF remains controversial. Our findings suggest a modest inverse association between tea intake and AF, which was primarily observed among individuals who consumed 2–3 cups of tea per day, whereas no statistically significant associations were observed for other intake categories. This is broadly consistent with some observational and experimental studies suggesting potential cardiovascular benefits of tea (16, 17). However, recent Mendelian randomization studies have not supported a causal effect of predicted tea consumption on cardiovascular diseases (18, 19), which differs from our observational findings. A variety of factors may contribute to these inconsistent results, such as differences in study design, outcome definitions coffee or tea consumption patterns in the study populations, imprecision due to relatively small numbers of AF cases, differences in exposure assessment, and duration of follow-up. Therefore, the tea findings should be interpreted more cautiously than the coffee findings.

Coffee and tea intake was associated with a lower risk of AF, the mechanism behind this protective effect is not fully understood, but there are several factors that may be involved. Firstly, the biological mechanisms underlying the observed associations remain uncertain and were not directly investigated in this study. One possible explanation is that coffee and tea contain polyphenols and flavonoids, which have been reported to possess anti-inflammatory and antioxidant properties (20, 21). Given that oxidative stress and inflammation have been implicated in the pathogenesis of AF (22), these bioactive compounds may contribute to the observed inverse associations. However, this hypothesis remains speculative because no biomarkers or mechanistic data were available in this study, and further biomarker-based and experimental studies are needed to clarify the underlying mechanisms. Secondly, it is important to note that our findings reflect associations with coffee and tea beverage consumption rather than with caffeine exposure itself. Because coffee intake in the UK Biobank included decaffeinated coffee and caffeine dose was not directly measured, the observed associations should not be interpreted as effects of caffeine alone. Nevertheless, caffeine remains one biologically plausible component of these beverages. Several studies have shown that moderate caffeine consumption may reduce the risk of AF (23). Caffeine acts as a stimulant to the central nervous system, promoting the release of neurotransmitters such as norepinephrine, which may affect cardiac function by improving myocardial contractility and electrical conduction (24).

This study has several strengths, including its large sample size, long follow-up, prospective design, and comprehensive adjustment for lifestyle and socioeconomic factors. However, several limitations should be acknowledged. Firstly, coffee and tea consumption was assessed only once at baseline. Beverage consumption patterns may change over the long follow-up period, and such changes could not be captured in this study, potentially leading to misclassification of long-term habitual intake and attenuation of the observed associations toward the null. Secondly, coffee and tea intake was based on self-reported data, which may be subject to recall bias and reporting error. Thirdly, cup size, brewing method, beverage strength, and preparation practices were not standardized, and caffeine dose was not directly measured. Because coffee intake included decaffeinated coffee, the exposure reflects overall beverage consumption rather than caffeine intake alone, which may have resulted in exposure misclassification. Fourthly, although we adjusted for multiple potential confounders, residual confounding may still exist because coffee and tea were analyzed in separate models without mutual adjustment, and unmeasured genetic, environmental, or other factors may also have influenced the results. Fifthly, due to data limitations, we were unable to examine the effects of different subtypes of coffee and tea on AF. Sixthly, because the study was conducted in the UK Biobank, our findings may be generalizable mainly to the UK population, and whether they can be extrapolated to other ethnic groups remains uncertain. Finally, as an observational study, our findings reflect associations rather than causal effects, and causal inference cannot be established from the present analyses.

## Conclusion

Our findings suggest that moderate coffee consumption, particularly 2–3 cups per day, was associated with a lower risk of AF. For tea, an inverse association was observed primarily among participants consuming 2–3 cups per day, and the magnitude of the association was modest. Further studies are necessary to confirm these findings in ethnically diverse populations and to elucidate the underlying biological mechanisms between coffee and tea intake and AF.

## Declarations

### Data availability

The data used in this study were obtained from the UK Biobank (https://www.ukbiobank.ac.uk/). UK Biobank data are available to approved researchers upon application. This research was conducted using UK Biobank application number 93398.
